# Proposed Comprehensive Methodology Integrated with Explainable Artificial Intelligence for Prediction of Possible Biomarkers in Metabolomics Panel of Plasma Samples for Breast Cancer Detection

**DOI:** 10.3390/medicina61040581

**Published:** 2025-03-25

**Authors:** Cemil Colak, Fatma Hilal Yagin, Abdulmohsen Algarni, Ali Algarni, Fahaid Al-Hashem, Luca Paolo Ardigò

**Affiliations:** 1 Department of Biostatistics, and Medical Informatics, Faculty of Medicine, Inonu University, Malatya 44280, Turkey; cemil.colak@inonu.edu.tr; 2 Department of Computer Science, King Khalid University, Abha 61421, Saudi Arabia; a.algarni@kku.edu.sa; 3 Department of Informatics and Computer Systems, College of Computer Science, King Khalid University, Abha 61421, Saudi Arabia; akafeer@kku.edu.sa; 4 Department of Physiology, College of Medicine, King Khalid University, Abha 61421, Saudi Arabia; fahaid999@yahoo.com; 5 Department of Teacher Education, NLA University College, Linstows Gate 3, 0166 Oslo, Norway

**Keywords:** breast cancer, metabolomics, biomarker, machine learning, explainable artificial intelligence

## Abstract

*Background and objectives*: Breast cancer (BC) is the most common type of cancer in women, accounting for more than 30% of new female cancers each year. Although various treatments are available for BC, most cancer-related deaths are due to incurable metastases. Therefore, the early diagnosis and treatment of BC are crucial before metastasis. Mammography and ultrasonography are primarily used in the clinic for the initial identification and staging of BC; these methods are useful for general screening but have limitations in terms of sensitivity and specificity. Omics-based biomarkers, like metabolomics, can make early diagnosis much more accurate, make tracking the disease’s progression more accurate, and help make personalized treatment plans that are tailored to each tumor’s specific molecular profile. Metabolomics technology is a feasible and comprehensive method for early disease detection and biomarker identification at the molecular level. This research aimed to establish an interpretable predictive artificial intelligence (AI) model using plasma-based metabolomics panel data to identify potential biomarkers that distinguish BC individuals from healthy controls. *Method and materials*: A cohort of 138 BC patients and 76 healthy controls were studied. Plasma metabolites were examined using LC-TOFMS and GC-TOFMS techniques. Extreme Gradient Boosting (XGBoost), Light Gradient Boosting Machine (LightGBM), Adaptive Boosting (AdaBoost), and Random Forest (RF) were evaluated using performance metrics such as Receiver Operating Characteristic-Area Under the Curve (ROC AUC), accuracy, sensitivity, specificity, and F1 score. ROC and Precision-Recall (PR) curves were generated for comparative analysis. The SHapley Additive Descriptions (SHAP) analysis evaluated the optimal prediction model for interpretability. *Results*: The RF algorithm showed improved accuracy (0.963 ± 0.043) and sensitivity (0.977 ± 0.051); however, LightGBM achieved the highest ROC AUC (0.983 ± 0.028). RF also achieved the best Precision-Recall Area under the Curve (PR AUC) at 0.989. SHAP search found glycerophosphocholine and pentosidine as the most significant discriminatory metabolites. Uracil, glutamine, and butyrylcarnitine were also among the significant metabolites. *Conclusions*: Metabolomics biomarkers and an explainable AI (XAI)-based prediction model showed significant diagnostic accuracy and sensitivity in the detection of BC. The proposed XAI system using interpretable metabolite data can serve as a clinical decision support tool to improve early diagnosis processes.

## 1. Introduction

Early diagnosis and treatment significantly increase survival rates for breast cancer (BC), one of the most common types of cancer in women worldwide [1]. The most widely used screening method is mammography; its sensitivity varies between 54% and 77%, and it has various limitations in early-stage tumor detection. Although the image resolution of mammography devices has improved with the advancement of digital technologies, there are significant difficulties in detecting tumors smaller than 5 mm [2,3]. Clinical practice is increasingly using alternative imaging modalities like thermography and magnetic resonance imaging (MRI) as complementary diagnostic tools to replace mammography. Still, these techniques may also have low sensitivity. This situation reveals the need for a new screening test that is non-invasive, highly sensitive, and specific [4]. However, significant metabolic transformations, primarily at the cellular level, support cancer cells’ ability to proliferate and resist signals that initiate cell death continuously. Unlike normal cells, cancer cells prefer different metabolic pathways that give them advantages in energy production and biosynthesis. Specific metabolite profiles define these transformations, particularly in cancerous tissues and circulating blood. Thus, the biochemical properties of tumors form unique cellular metabolic phenotypes [5,6,7,8].

While mammography remains a standard tool for BC screening, alternative methods like adjunctive ultrasonography, MRI, and contrast-enhanced mammography can improve sensitivity and detection rates, particularly in dense breasts. However, they may also have trade-offs in specificity and the potential for overestimation. In contrast, metabolomics has offered itself as a potential method for early cancer detection. Metabolomics profiling finds disease-specific metabolic phenotypes, mostly in plasma and tissue samples, by capturing the biochemical changes that only happen in cancer cells. The idea behind this study is that metabolomics with the help of artificial intelligence (AI) can reliably predict metabolic changes caused by cancer for the early detection and monitoring of BC. Metabolomics, an emerging field in precision oncology, comprehensively analyzes small molecule metabolites in biological systems. Recent advances in high-throughput technologies and computational methods have positioned metabolomics as a powerful tool for cancer biomarker discovery. In the last decade, with a better understanding of cancer at the molecular level, studies dealing with biomarker-based diagnostic methods have increased [9]. Biomarkers in metabolomics can be sensitive tools that can show whether someone has cancer by showing changes in metabolism that happen in cancerous tissues or the bloodstream. Metabolomics in the detection of disease involves the identification of biomarkers specific to every disease. Biomarkers offer early diagnosis and monitoring through changes in metabolic profiles [9,10].

Such complexities and high dimensionality in the structure of metabolomics data demand advanced computational methods during the analysis of the data [11]. AI techniques and machine learning have proven to be greatly successful in large-scale biological data analysis. Advanced AI approaches such as deep learning and ensembles have improved metabolomics data analysis by processing high-dimensional data with complicated interactions, detecting fine-level metabolic patterns missed by traditional statistical analysis, and merging multiple data types to enhance diagnostic power [12]. In particular, explainable AI (XAI) methods allow us to make predictions and understand the biological mechanisms behind these predictions. XAI also explains the underlying reasons for these predictions, increasing the reliability of model decisions and adding clinical interpretability. As an alternative to traditional black-box approaches, XAI helps us understand the feature dependencies of AI models, how they generate model predictions, and which variables play a dominant role in decision processes [13]. Bifarin et al. [14] used XAI to SHAP to study ovarian cancer serum metabolomics and renal cell carcinoma urine metabolomics. In the study, SHAP plots showed how metabolites interacted with each other, which helped find possible mechanistic relationships in people who had the different cancers. Novielli et al. [15] used the SHAP algorithm to figure out which colorectal cancer parameters were important for each person. They achieved this by assuming that there was a link between colorectal cancer and gut microbiome dysbiosis. The results identified specific bacteria such as Fusobacterium, Peptostreptococcus, and Parvimonas as biomarkers of colorectal cancer. By finding patterns in large numbers of data, like metabolomics data from plasma samples, XAI methods can help find specific biomarkers for disease diagnosis. Plasma samples provide an ideal biological material for being non-invasive and reflecting the general profile of circulating metabolites [16,17].

The main goal of this study is to create a prediction model that can find biomarker candidate metabolites that can tell the difference between people with BC and healthy people using a metabolomics platform that is based on plasma samples. For this purpose, an XAI model integrated with a tree-based machine learning approach was constructed in this study. Considering the importance of diagnosis with non-invasive methods in particular, analyzing circulating metabolite profiles is an important step in early diagnosis and treatment. In this context, the hypothesis of the study is that cancer-induced metabolic changes are distinct and measurable and that these changes can be reliably predicted by analyzing an XAI-supported model for the presence of BC. It is thought that the model to be developed will contribute to both the early diagnosis and the prediction of the course of the disease, thus improving the quality of life of individuals. The findings of this study will provide a new perspective on biomarker-based diagnostic approaches and make a valuable contribution to the field of cancer research.

## 2. Materials and Methods

### 2.1. Participants, Dataset, and Power Analysis

This study used metabolomics panel data based on plasma samples to develop an interpretable prediction model to distinguish BC from healthy controls. The study was conducted according to the principles of the Declaration of Helsinki and was approved by the Inonu University Health Sciences Non-Interventional Clinical Research Ethics Committee (protocol code = 2024/6682). The study data are in the Metabolomics Workbench database, an open-access data source, with the code ST000355. In the study, metabolomics data obtained from plasma samples of 138 patients diagnosed with BC and 76 healthy controls were examined. The median age of BC patients was 49.0 years (range, 31–73 years), while the median age of the healthy control group was 34.0 years (range, 21–40 years). According to TNM stage (Tumor, Node, Metastasis) distribution, 19 of the BC patients were TNM stage I, 50 were TNM stage II, 49 were TNM stage III, and 20 were TNM stage IV. In terms of hormone receptor status, 77 of the BC patients were estrogen receptor (ER) positive, 54 were negative, and seven were unknown. In terms of progesterone receptor (PR) status, 64 patients were positive, 67 patients were negative, and seven were unknown. HER-2 status was positive in 50 patients, negative in 80 patients, and unknown in eight patients (Table 1).

The BC was a patient group that did not include patients who were newly diagnosed, had relapsed, or were using any medication before sample collection. Control samples were collected from 76 healthy volunteers using the same sample collection protocol. Metabolite profiles in the samples were analyzed using liquid chromatography time-of-flight mass spectrometry (LC-TOFMS) and gas chromatography time-of-flight mass spectrometry (GC-TOFMS). High-resolution mass spectrometry techniques such as LC-TOFMS and GC-TOFMS provide a strong basis for the quantitative and qualitative analysis of metabolites by allowing for the sensitive detection of small molecules [18]. The sample size was calculated using the WSSPAS software [19] with α = 0.05, power = 0.80, and effect size = 0.5, requiring a minimum of 128 participants (64 per group). Our final sample exceeded this requirement with 138 BC patients and 76 controls.

### 2.2. Metabolomics Analysis

LC-TOFMS and GC-TOFMS techniques were utilized for metabolomics profiling due to their sensitivity and ability to detect low-abundance metabolites. Compared to traditional mass spectrometry, these methods offer superior accuracy in identifying and quantifying metabolites critical for biomarker discovery. In the sample preparation stage, 50 μL of plasma samples were labelled with two different internal standards (10 μL p-chlorophenyl alanine [0.1 mg/mL] and 10 μL heptadecanoic acid [1 mg/mL]) and extracted with 175 μL of methanol: chloroform (3:1) mixture. After being kept at −20 °C for 10 min, the samples were centrifuged at 13,000 rpm for 10 min. In GC-TOFMS analysis, samples were analyzed using Agilent 6890 N gas chromatography and Pegasus HT time-of-flight mass spectrometry system. Chromatographic separation was performed in the Rxi-5 ms capillary column (Crossbond 5% diphenyl/95% dimethyl polysiloxane) with a helium carrier gas (1.0 mL/min constant flow rate). In LC-TOFMS analysis, the Agilent HPLC 1200 system and a 4.6 × 150 mm 5 μm Agilent ZORBAX Eclipse XDB-C18 chromatography column were used. An Agilent model 6220 MSD TOF MS instrument was used for mass spectrometry analysis. Mass fragments were compared with the NIST 05 Standard mass spectral database and reference standards for metabolite identification. All blood samples were taken on an empty stomach in the morning and stored at −80 °C for two hours. A total of 225 metabolites were identified and quantified in metabolomics analyses. 102 (45.3%) of these metabolites were confirmed using reference standards (69 metabolites by GC-MS and 33 metabolites by LC-MS) [18].

### 2.3. Modelling and Explainable Artificial Intelligence Methodology

The metabolomics data obtained from these analyses were analyzed with statistical and AI approaches, and the aim was to determine BC-specific biomarkers. This study applied tree-based learning algorithms to increase prediction performance. Tree-based algorithms are powerful and flexible techniques used in classification and regression tasks. Modelling data through hierarchical decision structures provides high accuracy rates, especially on large data sets. In this study, eXtreme Gradient Boosting (XGBoost), Adaptive Boosting (AdaBoost), Light Gradient Boosting Machine (LightGBM), and Random Forest (RF) algorithms were used for BC diagnosis (Table 2). [20,21,22,23]. While Random Forest is an ensemble learning method that obtains more reliable results by combining the predictions of multiple decision trees, XGBoost is an advanced algorithm that corrects the errors of previous models at each step using the gradient boosting principle and provides high-performance optimization. AdaBoost is an algorithm that trains weak learners (usually simple decision trees) sequentially and creates a strong classifier by giving more weight to the misclassified examples at each step. At the same time, LightGBM is a gradient-boosting framework developed by Microsoft that provides a faster training time and low memory usage using a leaf-based growth strategy. AUC (Area Under the Curve) was the primary metric for evaluating model performance. Other performance metrics such as accuracy, sensitivity, specificity, F1 score, and Brier score were calculated, and the model’s success in each metric was evaluated according to the 10-fold cross-validation procedure. All performance metrics were expressed as mean and standard deviation, and the optimal model with the best prediction performance was determined according to the AUC results, which is the primary metric. Receiver Operating Characteristic (ROC) and Precision-Recall (PR) curves of the models were compared. SHapley Additive exPlanations (SHAP) analysis was applied to increase the interpretability of the best-performing model in the clinical decision-making process. Within the scope of this analysis, global feature importance, feature interactions, and SHAP dependency plots were obtained. Additionally, Spearmon rho correlation analysis was performed to examine the correlation coefficients between important biomarker candidate metabolites. All analyses were performed in the Python 3.9 programming language, and XGBoost, LightGBM, and SHAP packages were used based on the scikit-learn library. Relevant codes are located at https://github.com/drhilal/Breast-cancer-metabolomics.

### 2.4. Performance Evaluation of Machine Learning Models

A critical performance evaluation of our models has been obtained based on the latest metrics and reference literature for robust and reliable testing. The effectiveness of classifiers was tested with a complete set of metrics: AUC, which generalizes how well the model can distinguish between classes, plotting the true positive rate (TPR) against the false positive rate (FPR) across thresholds, and accuracy, expressing the correct classification rate for the model. Recall or Sensitivity is calculated, and it measures the number of actual positive cases correctly predicted. Specificity is the measurement of the percentage of actual negative cases correctly classified. The F1 score or the harmonic mean of Precision and Recall is achieved by balancing the change between these measurements. A well-crafted model means the estimated probability corresponds to the fact of the happening of the event. Virtually 80 percent of those women whose estimated probability of BC has been found at 0.8 would be classified as patients of BC. This is primarily for predictive models: clinical decision making has to see the model’s confidence in its own exact predictions. Therefore, we calibrate a trained model to obtain accurately forecasted probability, and the present article used the Brier score for the calibration in the final stage. The Brier score measures statistics applied to assess the probability forecasts’ accuracy. It is usually applied to probabilistic classification models. Brier scores evaluate the mean squared errors between the actual labels and predicted probabilities. The minimum value of the Brier score would suggest that predictions are closer to the real values [24,25].

The AUC estimates how well the model could discriminate between positive and negative instances at all levels. Its range is from 0 to 1, meaning the higher the value, the greater the discrimination. The AUC assesses how well the model performs in overall predictions of correct outcomes, such as in most significant medical decisions. An AUC of 0.5 denotes a stochastic model. AUC-PR was calculated, representing performance in terms of precision or positive predictive value and recall or sensitivity at various thresholds. The range of AUC PR is from 0 to 1. High values of AUC PR indicate good performance by the model. It is highly informative in imbalanced datasets because it measures the performance of the minority class, which is often the positive class. These metrics collectively ensure that the models are benchmarked vigorously and in standard fashion against criteria for evaluation [26,27].

### 2.5. Explainable Artificial Intelligence and SHapley Additive exPlanations

The scope of XAI can be delineated as local and global, achievable through the derivation of explanations for a single instance of the model or the entire model, respectively. Local explanations facilitate the interpretation of results for an individual patient. Clinicians can rely on the forecasts when the model’s explanations align with their thinking. The rationale can be conveyed to the patient regarding their specific instance, enhancing their confidence in applying machine learning models for diagnosis. Global explanations elucidate how the aggregate values of patient records impact the predictions generated by the model. These elucidations can be employed to discern patterns. Global explanations assist in recognizing model bias and extraneous features. These features may be omitted during further model training to reduce complexity. Although these explanations have numerous benefits, they also possess inherent limits. XAI frameworks cannot accurately describe the model and can only offer an approximation. This indicates that the non-linear correlation among the features may be too simplified [28]. XAI can be roughly classified into function-based and result-based techniques [29]. Function-based techniques clarify a model’s underlying mechanisms by analyzing its parameters, architecture, and decision-making processes. This technique includes concepts like Feature Importance, which evaluates the contribution of each feature to the predictions, thereby identifying the most important feature [30].

SHAP is an alternative explanatory framework that offers an interpretation of model predictions. The work [31] presented the application of SHAP values for assessing feature relevance and enhancing understanding of the model and its predictions. SHAP assigns a priority value to each characteristic for a particular output. The principal concept of SHAP is to compute the Shapley values for each feature within the dataset utilized for training and testing the machine-learning model intended for interpretation. Each Shapley value signifies the contribution of a feature in producing the model’s prediction. SHAP is based on the principle that each feature in the dataset has a corresponding Shapley value. SHAP is a method that is independent of any specific model. The principal disadvantage of Shapley values is that their computing complexity escalates exponentially, rendering them impractical as the number of features increases. This explains the emergence of approximations like Shapley Sampling Values and Kernel SHAP. Kernel SHAP requires reduced processing power to provide comparable accuracy [30,32].

## 3. Results

This study evaluated four machine-learning models (XGBoost, LightGBM, AdaBoost, and RF) for classifying BC metabolomics data. When the classification performances of the models were examined, the RF classifier achieved the highest accuracy (0.963 ± 0.043) and sensitivity (0.977 ± 0.051) values. However, the study determined AUC as the primary outcome, and the LightGBM model showed the best calibration with the highest AUC value (reached the lowest Brier score) (Table 3).

**Figure 1:**
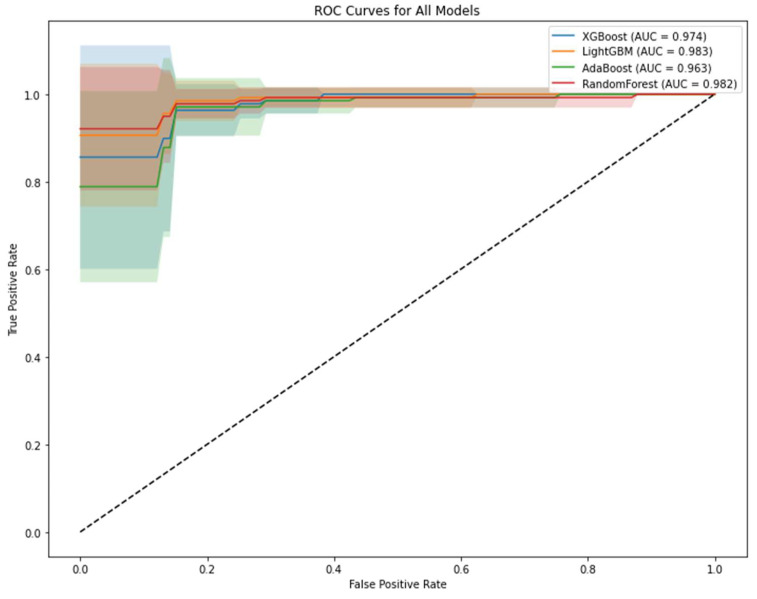
ROC curve comparison for tree-based models. LightGBM demonstrates the best discrimination with AUC = 0.983 ± 0.028, closely followed by Random Forest (AUC = 0.982 ± 0.030).

Figure 1 demonstrates ROC curve comparison for tree-based models. ROC curve analysis used to evaluate the discriminative performance of the models visualizes the True Positive Rate and False Positive Rate ratios at different threshold values. In the ROC analysis, LightGBM achieved the highest AUC value (0.983 ± 0.028), while RF (0.982 ± 0.030) and XGBoost (0.974 ± 0.045) also showed similar high performance. The proximity of the ROC curve to the upper left corner and the high AUC values indicate that the models have excellent classification performance.

**Figure 2:**
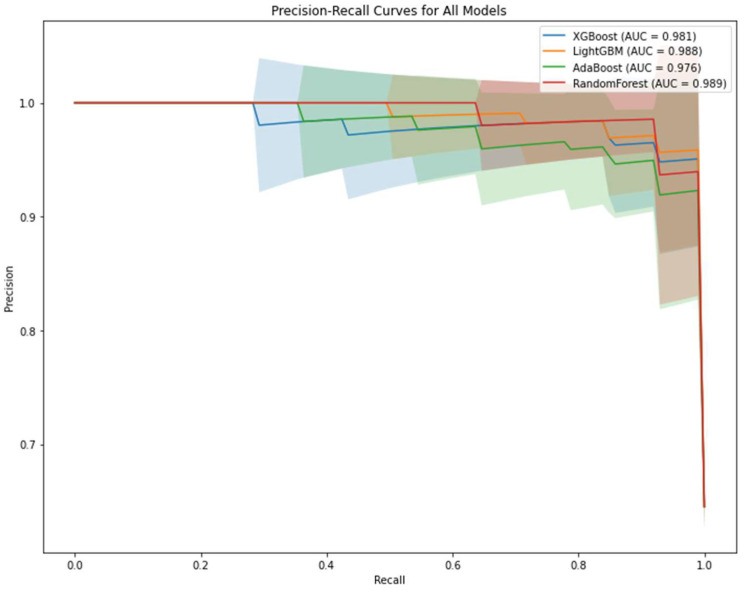
PR curve comparison, focusing on model performance with imbalanced datasets. Random Forest excels with AUC = 0.989, showcasing its robustness in detecting minority class instances.

Figure 2 illustrates the PR curve comparison of the predictive models. PR curves are used to evaluate the performance of the model, especially on imbalanced data sets. In the PR curve analysis, it is seen that RF has the highest AUC value (0.989), followed by LightGBM (0.988) and XGBoost (0.981). Maintaining high precision and recall values shows that the models are successful in both capturing true positives and minimizing false positives.

**Figure 3:**
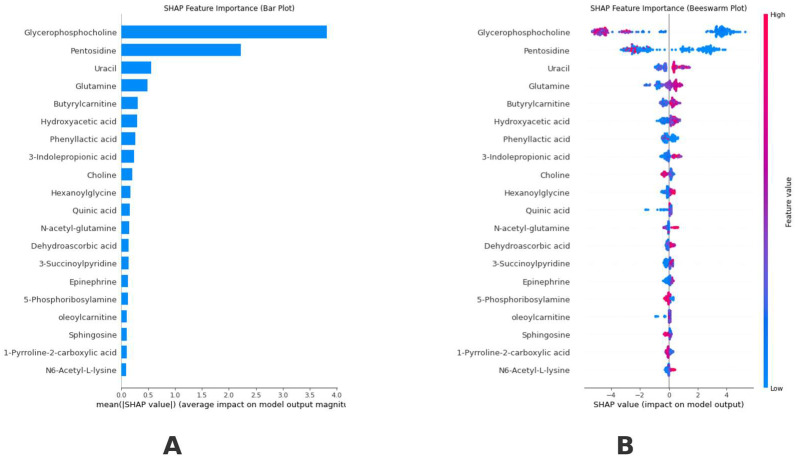
Visualizing model interpretability using explainable artificial intelligence tools. (**A**): Optimal model ranking of biomarker metabolites according to their relevance for clinical interpretation. (**B**): Average importance ranking (|SHAP value|) of notable biomarker metabolites; a greater SHAP value of a characteristic correlates with an increased likelihood of the patient being positive for BC.

Figure 3 visualizes model interpretability using explainable artificial intelligence tools. The SHAP analysis results used for model interpretability determined that Glycerophosphocholine and Pentosidine metabolites were the most effective biomarkers in model predictions in order of feature importance. The SHAP bar plot (Figure 3A) shows the average absolute contribution of each metabolite to the model predictions. The Beeswarm plot (Figure 3B) visualizes the effect of different values of each metabolite on the model output in detail; red dots represent high metabolite values, and blue dots represent low metabolite values. When the Beeswarm plot was examined, it was clearly observed that high values (red dots) of Glycerophosphocholine and Pentosidine metabolites were effective in positive class prediction. In contrast, low values (blue dots) effectively predicted a negative class. In addition, metabolites such as Uracil, Glutamine, and Butyrylcarnitine also played an important role in model predictions. The SHAP analysis results revealed a clear hierarchy of the effects of metabolites on the model output. Glycerophosphocholine was found to be the metabolite that had the strongest effect on the model predictions with an average SHAP value of 3.5 units. Pentosidine followed it with a SHAP value of approximately 2.0 units. Uracil and Glutamine showed moderate effects with SHAP values of approximately 0.5 units, respectively. In the Beeswarm plot analysis, it was observed that Glycerophosphocholine exhibited a biphasic effect pattern on the model output. It was observed that both high and low concentrations of this metabolite significantly affected the model predictions. A more linear effect pattern was found for Pentosidine, where it was determined that an increase in metabolite concentration positively affected the model output. The lower ranked metabolites (e.g., N6-Acetyl-L-lysine, 1-Pyrroline-2-carboxylic acid, Sphingosine) showed a minimal effect on the model predictions (SHAP values < 0.1). The distribution of these metabolites in the Beeswarm plot also confirms their limited effects; they cluster in a narrow range. These findings show that Glycerophosphocholine and Pentosidine metabolites play a dominant role in the model’s predictions. Thus, these metabolites can be potential biomarkers in the biological process examined.

**Figure 4:**
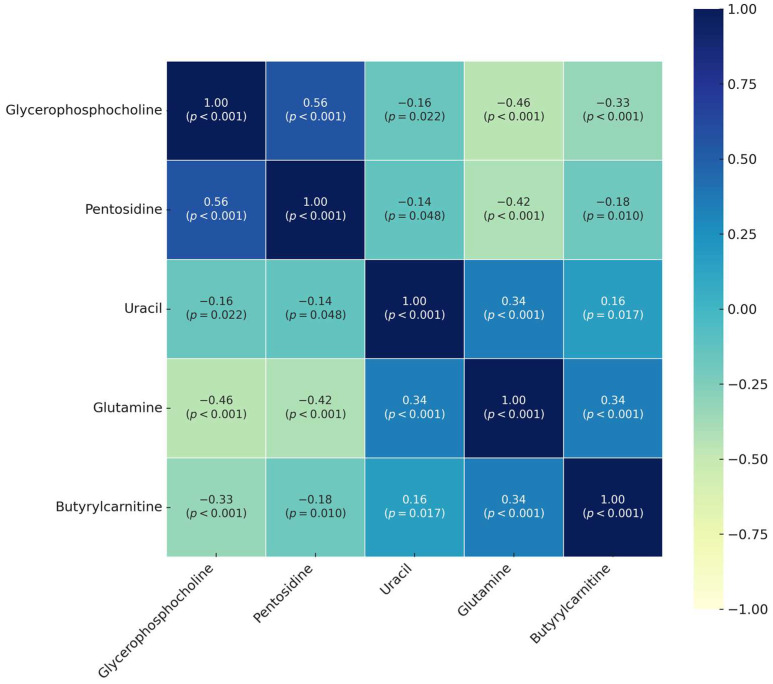
Correlation heatmap of the five most important metabolites in distinguishing breast cancer according to SHAP outputs.

Figure 4 depicts the correlation heatmap of the first five metabolites most important in distinguishing BC according to SHAP outputs. The prediction model received its most significant contribution from five metabolites consisting of Glycerophosphocholine, Pentosidine, Uracil, Glutamine, and Butyrylcarnitine based on the significance analysis of SHAP metabolite results. Glycerophosphocholine emerged as the top metabolite based on mean SHAP value because it proved to be the substance that influenced model output the most among all studied metabolites. Pentosidine displayed the second highest SHAP value. We tested assumed biological associations between the leading metabolites through correlation analysis. The data reveal a strong positive relationship between Glycerophosphocholine and Pentosidine, which resulted in a statistical value of rho 0.56 and *p* < 0.001, indicating possible co-accumulation and shared metabolic links between these metabolites. Research data indicated that Glycerophosphocholine possesses negative statistical relations to Glutamine (rho = −0.46, *p* < 0.001) and Butyrylcarnitine (rho = −0.33, *p* < 0.001), which implies adverse metabolic management. The metabolic results suggest that Glutamine positively affects both Uracil and Butyrylcarnitine metabolism, as indicated by statistical findings (rho = 0.34, *p* < 0.001).

## 4. Discussion

Although machine learning models hold great promise in BC prediction, their black-box nature remains a significant obstacle to their adoption in real-world scenarios [33,34]. A lack of interpretability and transparency may make medical professionals reluctant to use these models in real-world scenarios [35]. If a model predicts that a patient is at high risk for BC, the physician needs to know the reasons behind the prediction in order to make informed decisions about treatment and care. Therefore, black-box machine learning models can be potentially dangerous in some clinical and practical applications, i.e., when the models are actively used to recommend therapeutic actions. Therefore, in this study, SHAP, an XAI approach, was integrated into black-box machine learning models to distinguish BC from healthy controls and to discover important metabolomics biomarkers.

The results of this study highlight that metabolomics profiling has the potential to be used for BC diagnosis, and they demonstrate each step of how artificial intelligence methods can be applied to identify biomarkers. Our results demonstrate that circulating metabolites are essential information for BC diagnosis and that these biomarkers may be suitable clinical candidates as non-invasive diagnostic markers. These findings also receive similar supporting studies in the literature. Metabolomics technologies have been reported to hold great promise in cancer-related research by systematically describing metabolic biomarkers and pathways associated with BC diagnosis [36]. The combination of targeted and untargeted metabolomics platforms has been reported to provide a highly predictive and accurate method for early BC diagnosis from plasma samples [37]. It has been shown that metabolomics profiling can predict neurological and metabolic toxicity in BC treatment by considering environmental exposure, microbiota-related metabolites, and low-frequency metabolites [38]. These studies demonstrate the general diagnostic potential of metabolomics biomarkers, while in the context of BC these biomarkers can provide optimizing effects in early detection and treatment processes. In addition, it is suggested that using these biomarkers can optimize the effects in early detection and treatment processes of cancer [39,40].

In this study, we aimed to develop an XAI model integrated with tree-based machine learning to develop an interpretable prediction model using a plasma metabolomics dataset consisting of BC patients and healthy controls. In our analysis, we aimed to improve our understanding of BC predictors by examining metabolomics panel data and globally explained our model with strong discrimination using the SHAP approach. The current study determined that metabolites such as glycerophosphocholine and pentosidine were the most effective biomarkers in model predictions. The elevated levels of glycerophosphocholine in BC patients (SHAP value: 3.5) align with previous findings suggesting altered phospholipid metabolism in malignant transformation. Specifically, glycerophosphocholine elevation indicates increased membrane turnover; this metabolic shift may serve as an early indicator of malignant transformation, and monitoring glycerophosphocholine levels could potentially aid in treatment response assessment [41,42]. Based on the SHAP analysis, the changes in the levels of these metabolites affected positive and negative classifications in BC diagnosis. The results of SHAP values reported in this study show how each metabolite affects the prediction results and indicate the direction of change in the probability of BC classification with positive or negative values. The SHAP value for the glycerophosphocholine metabolite was calculated as 3.5, and this metabolite had the highest SHAP value. SHAP allows us to determine which features are most important within the model; however, the scale of measurement of SHAP values is not related to standard statistical units of measurement. Biologically, glycerophosphocholine is of high importance in biological processes, especially in phospholipid metabolism, which cancer cells use to promote rapid cell growth. Similarly, the association of Pentosidine with oxidative stress pathways highlights its potential relevance to tumor microenvironment dynamics. Clinical findings from earlier studies demonstrate that malignant transformations involving choline metabolism result in pathophysiological phosphocholine pathway dysregulation for tumor cell proliferation and resistance to apoptosis [43]. The evolution of glycerophosphocholine levels offers clinical information about disease advancement and therapy effectiveness because treatments focused on proliferative cells (such as chemotherapy) might stabilize phospholipid metabolic processes. Advanced glycation end-product pentosidine serves as an indicator for chronic inflammation combined with oxidative stress that exists in the tumor microenvironment. Its association with BC (SHAP value: 2.0) underscores the role of oxidative damage in oncogenesis. An elevated Pentosidine level indicates a higher production of reactive oxygen species (ROS) within cancer cells that results in genomic instability and promotes tumor-promoting signaling pathways. Medical reports indicate that the low measured variation in Pentosidine levels indicates its potential value as a reliable biomarker for monitoring oxidative stress effects on tumor behavior and therapy response [44,45]. BC research demonstrates that glycerophosphocholine and pentosidine analyze how metabolic reprogramming interacts with oxidative stress in BC. Through clinical workflow implementation, these biomarkers might improve both early diagnosis possibilities and treatment strategies made for individual patients.

The positive relationship between glycerophosphocholine and pentosidine (rho = 0.56, *p* < 0.001) may demonstrate the similar metabolic pathways of oxidative stress and protein glycation through which cell damage occurs. The current research showed that glycerophosphocholine had a negative correlation with glutamine levels (rho = −0.46, *p* < 0.001). Metabolic and nitrogen transfer functions combine with nucleotide biosynthesis and redox regulation as major functions of the essential amino acid glutamine. The simultaneous reduction of glutamine levels with corresponding increases in glycerophosphocholine indicates metabolic changes directed toward augmented membrane deterioration, which may relate to cellular stress or energy requirement adjustments. Glycerophosphocholine displays a negative correlation with butyrylcarnitine (rho = −0.33, *p* < 0.001), which is a short-chain acylcarnitine that potentially influences mitochondrial β-oxidation by way of any membrane system disorders. Glutamine showed positive correlations with the levels of both uracil (rho = 0.34, *p* < 0.001) and butyrylcarnitine (rho = 0.34, *p* < 0.001), which may indicate that amino acid metabolism coordinates with nucleotide and energy metabolism. The levels of uracil may increase due to enhanced activities of nucleic acid breakdown processes or cell multiplication or tissue injuries. Glutamine together with butyrylcarnitine in the body demonstrates a relationship that connects mitochondrial roles between energy metabolism regulation and nitrogen balance maintenance. The results from the correlation study prove that SHAP findings have strong biological validity while discovering relationships that explain how metabolic processes generate model prediction results. Membrane lipid degradation, together with oxidative stress and amino acid depletion and impaired mitochondrial function, appears to build the main metabolic markers behind BC development.

This study points to the clinical significance of the biomarkers of glycerophosphocholine and pentosidine in diagnosing BC, for which they may be applicable. The findings suggest that these metabolites provide promising opportunities to diagnose and monitor BC. The role of glycerophosphocholine in reflecting changes in cell membrane metabolism is particularly important. According to a medical study [43], the naturally elevated levels of phosphocholine and relatively lower levels of glycerophosphocholine in aggressive BC cells make it an important biomarker. Furthermore, glycerophosphodiester phosphodiesterase domain containing five was also identified as a regulator for choline phospholipid metabolism and proliferated the known critical role of this metabolic pathway in BC. The relationship between pentosidine and oxidative stress and inflammation opens a new window in understanding the tumor microenvironment. The low coefficients of variation (6.5% and 2.0%) shown in the study by Scheijen et al. [44] support the reliability of this biomarker. This finding is important for the usability of pentosidine in clinical applications. The contribution of the metabolomics approach in this area cannot be ignored. Measuring the composition of small molecules in tissue, blood, or urine provides important contributions to the understanding of the disease at the molecular level [45]. In light of these findings, larger-scale clinical studies are needed to evaluate the usability of glycerophosphocholine and pentosidine in the diagnosis and follow-up of BC. It is important to investigate the value of these biomarkers, especially in different patient subgroups and at different stages of the disease.

Significant contributors to the predictive model’s performance are metabolites, including uracil, glutamine, and butyrylcarnitine, indicating metabolomic biomarkers’ feasibility in early diagnosis and disease monitoring [46,47].

Refs. [48,49]. Our findings are consistent with previous studies describing the importance of metabolomic biomarkers in progressing BC and boosting predictive models. The metabolic pathways that contribute to cancer cell proliferation, metastasis, and resistance to treatment alter uracil, glutamine, and butyrylcarnitine. If these processes are understood, it may help point towards targeted therapies and more accurate prognostic models. In advanced BC stages, malignant cell growth, metastasis, and drug resistance depend on glutamine metabolism. For example, mTOR is a critical mediator of glutamine metabolism in hormone receptor positive BC that heavily depend on glutamine metabolism. The inhibition of tumor growth by targeting glutamine metabolism, particularly the Glutaminase enzyme, has been promising. Glutamine metabolism, critical for advanced-stage BC, supports cell proliferation and drug resistance. Studies highlight mTOR’s role in glutamine-driven pathways, with glutaminase inhibitors showing therapeutic potential [50].

Ref. [51]. Our findings align with prior work using metabolomic profiling to establish glutamine as a pivotal biomarker in BC diagnostics. Glutamine metabolomic profiling was critical in constructing prognostic models for BC. Metabolic genes are included in these models and validated across multiple datasets, lending weight to their potential clinical application. Glutamine was another key biomarker identified as being important for early BC diagnosis via a study with 1H-NMR technology. Both uracil and butyrylcarnitine are documented to be part of the BC metabolic profile and may work together to modulate disease progression or treatment outcome. Combining multiple metabolites into a model allows for a more comprehensive understanding of BC biology, enabling the construction of personalized treatment strategies. Additional studies of the roles of uracil and butyrylcarnitine could identify new therapeutic targets and yield additional avenues for novel treatment interventions diagnosis and disease monitoring [46,47,48,49].

Further strengthening the results of this study are the ROC and PR analyses used in the evaluation of the performances of the models. Among the compared models, the highest AUC value obtained in the ROC analysis was from the LightGBM model, while in the PR analysis, the highest AUC value was obtained from the RF model. In both analyses, it was determined that these models showed excellent classification performance. These findings comply with similar studies in the literature. It has been reported that LightGBM outperformed RF in terms of classification performance in metabolomics data. However, RF produces a good value compared to LightGBM on balanced data, while LightGBM has been shown to successfully handle unbalanced data [51]. In addition, RF remains a robust choice for balanced datasets, offering high interpretability and resistance to overfitting, which may be advantageous in certain analytical contexts. These studies also pointed out the importance of model selection, especially for the multidimensional structure of metabolomics data, and supported the findings of this study. However, the specific advantages of different models also revealed that more in-depth research is needed, especially on unbalanced datasets [52].

It is also noteworthy that metabolomics analysis is changing our view of what impact it can have on clinical decision-making processes. This study showed that plasma metabolite profiles, a noninvasive method, are much more sensitive and specific than traditional diagnostic methods. This demonstrates the feasibility of a novel diagnostic tool to complement mammography and other traditional techniques with sensitivity and specificity deficiencies [53].

The limitations of traditional methods such as mammography, whose sensitivity and specificity are often lower in dense breast tissue, are striven to be rectified in the development of new diagnostic tools that enhance BC detection. Improving accuracy while increasing accessibility is a primary goal of recent advances in deep learning and computer-aided diagnosis (CAD) systems. Recent work has demonstrated great potential in developing CNNs to perform high-accuracy BC detection using advanced image processing approaches, such as YCbCr color space, for enhanced classification [54].

Applying Tchebichef features and optimized extreme learning machines in a novel CAD system for mammogram classification achieved 100% accuracy on average with no distribution dependence and is an effective auxiliary tool for radiologists [53].

Despite the enormous potential of these innovative tools, their widespread use in low-resource settings presents challenges due to limited access to cutting-edge devices. Comparable studies in the literature also support this potential of metabolomics biomarkers. Nevertheless, integrating metabolism biomarkers into clinical practice is a challenging process that requires a consideration of various factors, such as standardized measurement, information technology infrastructure, and cost analysis. Establishing standard operating procedures for collecting and processing samples, implementing quality controls, developing clinical bioinformatics tools, and fostering teamwork among disciplines are of utmost importance. The lack of regulatory frameworks and the need for multidisciplinary work also raise concerns about resource optimization [51,52].

This makes the early economic evaluation of new biomarkers and the analysis of their financial impacts in clinical practice crucial [55,56]. This study also has some limitations. The first issue is that the data set’s size may not be sufficiently extensive to generalize to the general population, given its uneven distribution. Furthermore, the study’s design is retrospective, which does not allow for an external validation of the results obtained using prospective studies. Although we avoided reporting biased results by using 10-fold cross-validation as a validation procedure for the models, another limitation is that the study’s results could not be validated on an independent dataset to demonstrate the robustness of the models. Future studies should validate the current results with an external validation set, and using larger and more diverse datasets can generalize these findings. In addition, future studies may include a prospective cohort study recruiting newly diagnosed BC patients and healthy controls in a clinical setting. This will allow plasma metabolomics data to be collected using standard protocols to assess the predictive performance of our models in real-time patient diagnosis. Additionally, longitudinal follow-up data may be included to determine whether metabolomics changes can predict treatment response and disease progression. This prospective validation will provide further evidence to support the clinical integration of metabolomics-based biomarkers in BC screening and early diagnosis. Additionally, in this study, the importance of plasma metabolomics biomarkers (e.g., glycerophosphocholine and pentosidine) for the early diagnosis of BC was highlighted by SHAP analysis. While high levels of glycerophosphocholine were associated with malignant transformation (SHAP value: 3.5), Pentosidine was reported to have low coefficients of variation associated with oxidative stress. Even though methods like LC-TOFMS and GC-TOFMS are very sensitive, there are not any exact concentration ranges or detection limits for biomarkers. It is recommended to validate these parameters by targeted metabolomics in further studies [18,44]. Future studies should strengthen SHAP-derived feature interpretations by integrating statistical validation methods such as bootstrapping (confidence intervals), permutation tests (significance, *p*-values), and so on to align metabolite rankings with clinical outcomes. Additionally, future research should quantify the detection limits (e.g., LOD/LOQ) of plasma-derived biomarkers (e.g., glycerophosphocholine, pentosidine) using targeted assays and validate their BC specificity through cross-cancer cohort comparisons to ensure clinical applicability.

In conclusion, the current study has demonstrated the potential use of metabolomic biomarkers in BC diagnosis and the advantages of artificial intelligence-supported models. This study illustrates the potential for AI-integrated metabolomics for diagnosing breast cancer and identifying key biomarkers of glycerophosphocholine and pentosidine. Both these findings strongly implicate noninvasive, precise diagnostic tools and suggest that they warrant large-scale validation studies before clinical applicability. In the literature, studies on the use of metabolomics biomarkers in early diagnosis and disease management in different types of cancer offer similarly promising results. However, further studies are required to analyze metabolomics data and make biomarkers suitable for clinical use. In particular, advanced studies on large data sets in different populations will increase the generalizability and effectiveness of biomarkers. Integrating interdisciplinary approaches and innovative technologies can further accelerate progress in this field. In addition, the applicability of these biomarkers in healthcare can be strengthened by developing clinical decision support systems.

## Figures and Tables

**Table 1:** Descriptive statistics of study participants.

Characteristic	Breast Cancer	Healthy Control
Number of Participants	138	76
Age (median, range)	49.0 (31–73)	34.0 (21–40)
TNM Stage I	19	
TNM Stage II	50	
TNM Stage III	49	
TNM Stage IV	20	
ER (Positive/Negative/Unknown)	77/54/7	
PR (Positive/Negative/Unknown)	64/67/7	
HER-2 (Positive/Negative/Unknown)	50/80/8	

**Table 2:** Definitions of machine learning models utilized in this study.

Model	Type	Description
RF	Ensemble ML; Bagging	Random Forest is an ensemble technique that generates several decision trees during the training phase and produces the mode of the classes for classification purposes. By averaging several deep decision trees trained on different parts of the same training dataset, it improves the accuracy of predictions and reduces overfitting.
AdaBoost	Ensemble ML; Boosting	AdaBoost is an enhancement method that integrates weak learners to form a robust classifier. It assigns weights to misclassified instances and modifies them in subsequent iterations to emphasize difficult-to-classify occurrences, thereby enhancing the model’s accuracy.
LightGBM	Ensemble ML; Boosting	LightGBM is a gradient-boosting framework that emphasizes the efficiency of model construction. It employs methods such as histogram-based learning and exclusive feature bundling to accelerate training and enhance performance, particularly on extensive datasets.
XGBoost	Ensemble ML; Boosting	XGboost is a sophisticated version of gradient boosting that employs a regularized model structure to mitigate overfitting. It has sophisticated capabilities like parallel processing, tree pruning, and the management of missing information, rendering it rapid and effective for structured data analysis.

**Table 3:** Performance metrics of machine learning models.

Metric	XGBoost	LightGBM	AdaBoost	Random Forest
Accuracy	0.949 ± 0.056	0.949 ± 0.046	0.939 ± 0.071	0.963 ± 0.043
Sensitivity	0.948 ± 0.080	0.956 ± 0.062	0.949 ± 0.084	0.977 ± 0.051
Specificity	0.948 ± 0.067	0.934 ± 0.070	0.920 ± 0.097	0.934 ± 0.091
F1	0.958 ± 0.048	0.959 ± 0.038	0.951 ± 0.056	0.971 ± 0.034
AUC	0.974 ± 0.045	0.983 ± 0.028	0.963 ± 0.043	0.982 ± 0.030
Brier	0.044 ± 0.044	0.034 ± 0.036	0.134 ± 0.024	0.069 ± 0.022

XGBoost: Extreme gradient boosting; LightGBM: Light gradient boosting machine; AdaBoost: Adaptive boosting; AUC: Area under the curve.

## Data Availability

Data can be requested from the corresponding author upon appropriate request.

## Abbreviations List

AI	Artificial Intelligence
AUC	Area Under the Curve
PR-AUC	Precision-Recall Area Under the Curve
BC	Breast Cancer
CAD	Computer-Aided Diagnosis
CNN	Convolutional Neural Network
ER	Estrogen Receptor
GC-TOFMS	Gas Chromatography-Time of Flight Mass Spectrometry
HER-2	Human Epidermal Growth Factor Receptor 2
LC-TOFMS	Liquid Chromatography-Time of Flight Mass Spectrometry
LightGBM	Light Gradient Boosting Machine
mTOR	Mechanistic Target of Rapamycin
MRI	Magnetic Resonance Imaging
NMR	Nuclear Magnetic Resonance
PC	Phosphocholine
RF	Random Forest
ROC	Receiver Operating Characteristic
ROS	Reactive Oxygen Species
SHAP	SHapley Additive exPlanations
TNM	Tumor, Node, Metastasis
TOFMS	Time-of-Flight Mass Spectrometry
XAI	Explainable Artificial Intelligence
XGBoost	eXtreme Gradient Boosting

## References

[1] Smolarz B., Nowak A.Z., Romanowicz H.J.C. (2022). Breast cancer—Epidemiology, classification, pathogenesis and treatment (review of literature). Cancers.

[2] Skaane P. (2009). Studies comparing screen-film mammography and full-field digital mammography in breast cancer screening: Updated review. Acta Radiol..

[3] Böhm D., Keller K., Wehrwein N., Lebrecht A., Schmidt M., Kölbl H., Grus F.-H. (2011). Serum proteome profiling of primary breast cancer indicates a specific biomarker profile. Oncol. Rep..

[4] Sree S.V., Ng E.Y.-K., Acharya U R., Tan W. (2010). Breast imaging systems: A review and comparative study. J. Mech. Med. Biol..

[5] Zhu J., Thompson C.B. (2019). Metabolic regulation of cell growth and proliferation. Nat. Rev. Mol. Cell Biol..

[6] Kalyanaraman B. (2017). Teaching the basics of cancer metabolism: Developing antitumor strategies by exploiting the differences between normal and cancer cell metabolism. Redox Biol..

[7] Alwahsh M., Abumansour H., Althaher A.R., Hergenröder R. (2024). Metabolic Profiling Techniques and their Application in Cancer Research. Curr. Pharm. Anal..

[8] Martínez-Reyes I., Chandel N.S. (2021). Cancer metabolism: Looking forward. Nat. Rev. Cancer.

[9] Muthubharathi B.C., Gowripriya T., Balamurugan K. (2021). Metabolomics: Small molecules that matter more. Mol. Omics.

[10] Sarhadi V.K., Armengol G. (2022). Molecular biomarkers in cancer. Biomolecules.

[11] Ren S., Hinzman A.A., Kang E.L., Szczesniak R.D., Lu L.J. (2015). Computational and statistical analysis of metabolomics data. Metabolomics.

[12] Guldogan E., Yagin F.H., Pinar A., Colak C., Kadry S., Kim J. (2023). A proposed tree-based explainable artificial intelligence approach for the prediction of angina pectoris. Sci. Rep..

[13] Cansel N., Hilal Yagin F., Akan M., Ilkay Aygul B. (2023). Interpretable estimation of suicide risk and severity from complete blood count parameters with explainable artificial intelligence methods. Psychiatr. Danub..

[14] Bifarin O.O., Fernández F.M. (2024). Automated machine learning and explainable AI (AutoML-XAI) for metabolomics: Improving cancer diagnostics. J. Am. Soc. Mass Spectrom..

[15] Novielli P., Romano D., Magarelli M., Bitonto P.D., Diacono D., Chiatante A., Lopalco G., Sabella D., Venerito V., Filannino P. (2024). Explainable artificial intelligence for microbiome data analysis in colorectal cancer biomarker identification. Front. Microbiol..

[16] Irajizad E., Wu R., Vykoukal J., Murage E., Spencer R., Dennison J.B., Moulder S., Ravenberg E., Lim B., Litton J. (2022). Application of artificial intelligence to plasma metabolomics profiles to predict response to neoadjuvant chemotherapy in triple-negative breast cancer. Front. Artif. Intell..

[17] Li N., Yang C., Zhou S., Song S., Jin Y., Wang D., Liu J., Gao Y., Yang H., Mao W. (2021). Combination of plasma-based metabolomics and machine learning algorithm provides a novel diagnostic strategy for malignant mesothelioma. Diagnostics.

[18] Xie G., Zhou B., Zhao A., Qiu Y., Zhao X., Garmire L., Shvetsov Y.B., Yu H., Yen Y., Jia W. (2015). Lowered circulating aspartate is a metabolic feature of human breast cancer. Oncotarget.

[19] Arslan A.K., Yaşar Ş., Çolak C., Yoloğlu S. (2018). WSSPAS: An interactive web application for sample size and power analysis with R using shiny. Türkiye Klin. Biyoistatistik.

[20] Chen T., Guestrin C. Xgboost: A scalable tree boosting system. Proceedings of the 22nd ACM SIGKDD International Conference on Knowledge Discovery and Data Mining.

[21] Freund Y., Schapire R.E. (1997). A decision-theoretic generalization of on-line learning and an application to boosting. J. Comput. Syst. Sci..

[22] Ke G., Meng Q., Finley T., Wang T., Chen W., Ma W., Ye Q., Liu T.-Y. (2017). Lightgbm: A highly efficient gradient boosting decision tree. Adv. Neural Inf. Process. Syst..

[23] Breiman L. (2001). Random forests. Mach. Learn..

[24] Huang Y., Li W., Macheret F., Gabriel R.A., Ohno-Machado L. (2020). A tutorial on calibration measurements and calibration models for clinical prediction models. J. Am. Med. Inform. Assoc..

[25] Liu J., Wang C., Yan R., Lu Y., Bai J., Wang H., Li R. (2022). Machine learning-based prediction of postpartum hemorrhage after vaginal delivery: Combining bleeding high risk factors and uterine contraction curve. Arch. Gynecol. Obstet..

[26] Saito T., Rehmsmeier M. (2015). The precision-recall plot is more informative than the ROC plot when evaluating binary classifiers on imbalanced datasets. PLoS ONE.

[27] Ozenne B., Subtil F., Maucort-Boulch D. (2015). The precision–recall curve overcame the optimism of the receiver operating characteristic curve in rare diseases. J. Clin. Epidemiol..

[28] Petch J., Di S., Nelson W. (2022). Opening the black box: The promise and limitations of explainable machine learning in cardiology. Can. J. Cardiol..

[29] Saranya A., Subhashini R. (2023). A systematic review of Explainable Artificial Intelligence models and applications: Recent developments and future trends. Decis. Anal. J..

[30] Nasir Y., Kadian K., Sharma A., Dwivedi V. (2024). Interpretable machine learning for dermatological disease detection: Bridging the gap between accuracy and explainability. Comput. Biol. Med..

[31] Scott M., Su-In L.J.A. (2017). A unified approach to interpreting model predictions. Neural Inf. Process. Syst..

[32] Aas K., Jullum M., Løland A. (2021). Explaining individual predictions when features are dependent: More accurate approximations to Shapley values. Artif. Intell..

[33] Hassija V., Chamola V., Mahapatra A., Singal A., Goel D., Huang K., Scardapane S., Spinelli I., Mahmud M., Hussain A. (2024). Interpreting black-box models: A review on explainable artificial intelligence. Cogn. Comput..

[34] Vimbi V., Shaffi N., Mahmud M. (2024). Interpreting artificial intelligence models: A systematic review on the application of LIME and SHAP in Alzheimer’s disease detection. Brain Inform..

[35] Tasnim N., Al Mamun S., Shahidul Islam M., Kaiser M.S., Mahmud M. (2023). Explainable mortality prediction model for congestive heart failure with nature-based feature selection method. Appl. Sci..

[36] Yang L., Wang Y., Cai H., Wang S., Shen Y., Ke C. (2020). Application of metabolomics in the diagnosis of breast cancer: A systematic review. J. Cancer.

[37] Wei Y., Jasbi P., Shi X., Turner C., Hrovat J., Liu L., Rabena Y., Porter P., Gu H. (2021). Early breast cancer detection using untargeted and targeted metabolomics. J. Proteome Res..

[38] Piffoux M., Jacquemin J., Pétéra M., Durand S., Abila A., Centeno D., Joly C., Lyan B., Martin A.-L., Everhard S. (2024). Metabolomic Prediction of Breast Cancer Treatment–Induced Neurologic and Metabolic Toxicities. Clin. Cancer Res..

[39] Díaz-Beltrán L., González-Olmedo C., Luque-Caro N., Díaz C., Martín-Blázquez A., Fernández-Navarro M., Ortega-Granados A.L., Gálvez-Montosa F., Vicente F., Pérez del Palacio J.P. (2021). Human plasma metabolomics for biomarker discovery: Targeting the molecular subtypes in breast cancer. Cancers.

[40] Haince J.-F., Zhang L., Bux R.A., Tappia P.S., Ramjiawan B., Wishart D., Maksymiuk A. (2024). Abstract PO5-13-03: Early Detection of Breast Cancer using Targeted Plasma Metabolomic Profiling. Cancer Res..

[41] Sterin M., Cohen J.S., Mardor Y., Berman E., Ringel I. (2001). Levels of phospholipid metabolites in breast cancer cells treated with antimitotic drugs: A 31P-magnetic resonance spectroscopy study. Cancer Res..

[42] Stoica C., Ferreira A., Hannan K., Bakovic M. (2022). Bilayer Forming Phospholipids as Targets for Cancer Therapy. Int. J. Mol. Sci..

[43] Wijnen J., Jiang L., Greenwood T., Cheng M., Döpkens M., Cao M., Bhujwalla Z., Krishnamachary B., Klomp D., Glunde K. (2014). Silencing of the glycerophosphocholine phosphodiesterase GDPD5 alters the phospholipid metabolite profile in a breast cancer model in vivo as monitored by 31P MRS. NMR Biomed..

[44] Scheijen J.L., van de Waarenburg M.P., Stehouwer C.D., Schalkwijk C.G. (2009). Measurement of pentosidine in human plasma protein by a single-column high-performance liquid chromatography method with fluorescence detection. J. Chromatogr. B.

[45] Günther U.L. (2015). Metabolomics biomarkers for breast cancer. Pathobiology.

[46] Demas D.M., Demo S., Fallah Y., Clarke R., Nephew K.P., Althouse S., Sandusky G., He W., Shajahan-Haq A.N. (2019). Glutamine metabolism drives growth in advanced hormone receptor positive breast cancer. Front. Oncol..

[47] Wang Y., Zhang H., Chu Y. (2023). Advances in Research on the Relationship between Glutamine Metabolism and Breast Cancer. Int. J. Biol. Life Sci..

[48] Liu Y., Qi C., Zheng L., Li J., Wang L., Yang Y. (2022). 1 H-NMR based metabolic study of MMTV-PyMT mice along with pathological progress to screen biomarkers for the early diagnosis of breast cancer. Mol. Omics.

[49] Yu R., Peng M., Zhao S., Wang Z., Ma Y., Zhang X., Lv X., Wang S., Ju S., Zhao R. (2022). Comprehensive Characterization of the Function of Metabolic Genes and Establishment of a Prediction Model in Breast Cancer. Dis. Markers.

[50] Mirshekaran R., Ahmadi K., Shahbazi B., Farshidfar G., Eftekhar E., Kavousipour S. (2024). Cancer Therapy Potential Unveiled: FDA-Approved Drugs Targeting Glutaminase for Breast Cancer Treatment. ChemistrySelect.

[51] Sari L., Romadloni A., Lityaningrum R., Hastuti H.D. (2023). Implementation of LightGBM and Random Forest in Potential Customer Classification. TIERS Inf. Technol. J..

[52] Duran F., Wijaya F., Hulu Y.R., Harahap M., Prabowo A. (2023). Perbandingan Kinerja Algoritma Random Forest Classifier Dan Lightgbm Classifier Untuk Prediksi Penyakit Jantung. Data Sci. Indones. (DSI).

[53] Mohanty F., Rup S., Dash B., Majhi B., Swamy M.N.S. (2019). A computer-aided diagnosis system using Tchebichef features and improved grey wolf optimized extreme learning machine. Appl. Intell..

[54] Hamid M.A., Mondher H.M., Ayoub B. Deep Learning CNNs for Breast Cancer Classification and Detection” Enhancing Diagnostic Accuracy in Medical Practice. Proceedings of the 2024 2nd International Conference on Electrical Engineering and Automatic Control (ICEEAC).

[55] Long N.P., Nghi T.D., Kang Y.P., Anh N.H., Kim H.M., Park S.K., Kwon S.W. (2020). Toward a standardized strategy of clinical metabolomics for the advancement of precision medicine. Metabolites.

[56] Marchand C.R., Farshidfar F., Rattner J., Bathe O.F. (2018). A framework for development of useful metabolomic biomarkers and their effective knowledge translation. Metabolites.

